# The enhanced activity of Pt–Ce nanoalloy for oxygen electroreduction

**DOI:** 10.1038/s41598-020-71965-0

**Published:** 2020-09-09

**Authors:** Juan Qin, Yafeng Zhang, Deying Leng, Feng Yin

**Affiliations:** 1 School of Physics and Information Technology, Shaanxi Normal University, Xi’an, 710119 China; 2 Key Laboratory of Syngas Conversion of Shaanxi Province, Shaanxi Normal University, Xi’an, 710119 China

**Keywords:** Environmental sciences, Energy science and technology, Materials science, Nanoscience and technology

## Abstract

The widespread use of low-temperature polymer electrolyte membrane fuel cells for clean energy source require significant reductions in the amount of expensive electrocatalyst Pt for the oxygen reduction reaction (ORR). Pt based binary alloys are promising materials for more active and stable electrocatalysts. In this paper, we studied Pt–Ce nanoalloy, which was prepared by hydrogen reduction techniques as ORR electrocatalysts. Among all PtCe alloy catalysts, the PtCe/C-800 ℃ shows superior ORR activity, stability and durability compared to commercial Pt/C. The results presented in this paper will provide the future perspectives to research based on Pt-RE (RE = Ce, Dy, Gd, Er, Sm, and La) alloy as an novel electrocatalyst for various electrocatalytic reactions.

## Introduction

The low-temperature polymer electrolyte membrane fuel cells (PEMFCs) are promising alternative devices for clean energy source. For PEMFCs to become economically viable, several problems must be overcome. One of the pivotal issue is the over potential associated with the slow kinetics of the oxygen reduction reaction (ORR: O_2_ + 4H^+^ + 4e^−^ = 2H_2_O) at the cathode. Up to now, Pt is the most widely used electrocatalyst for ORR. However, the high cost and low storage of Pt strongly limit the expansion of PEFCs^1–3^. In recent years, the employment of Pt alloys with transition metals (Ni, Cu and Co etc.) has attracted wide attention due to it obviously reduces Pt content and shows significant improvements in ORR activity over pure Pt^4–11^. But Pt/transition metal alloy displays poor stability for ORR due to the transition metal tends to dissolve in the acidic electrolyte of PEMFC. Recent reports on Pt/rare earth element alloy electrocatalysts demonstrate that the doping of Pt with rare earth elements can effectively avoid the dealloying and exhibit high ORR activity^12–20^. It was considered that the alloying energy, or the negative enthalpy of formation retards the dissolution of rare element from the alloy. Chemical synthesis of Pt/rare-earth nanoalloy is still a real challenge, partially because of the oxophilicity of rare-earth elements. So research in this area has focused mainly on both polycrystalline and single-crystal electrodes partially.

In this paper, we describe method for making Pt–Ce nanoalloy electrocatalysts using hydrogen reduction technique. In order to provide insights and understanding on the structure and property of Pt–Ce nanoalloy electrocatalyst, the Pt–Ce nanoalloy samples were characterized and analyzed by several techniques, including transmission electron microscopy (TEM) with energy dispersive X-ray spectroscopy (EDX), X-ray diffraction (XRD), inductively coupled plasma optical emission spectrometer (ICP-OES), X-ray photoelectron spectroscopy (XPS), cyclic voltammetry (CV) and chronoamperometry (CA).

## Results and discussion

**Figure 1:**
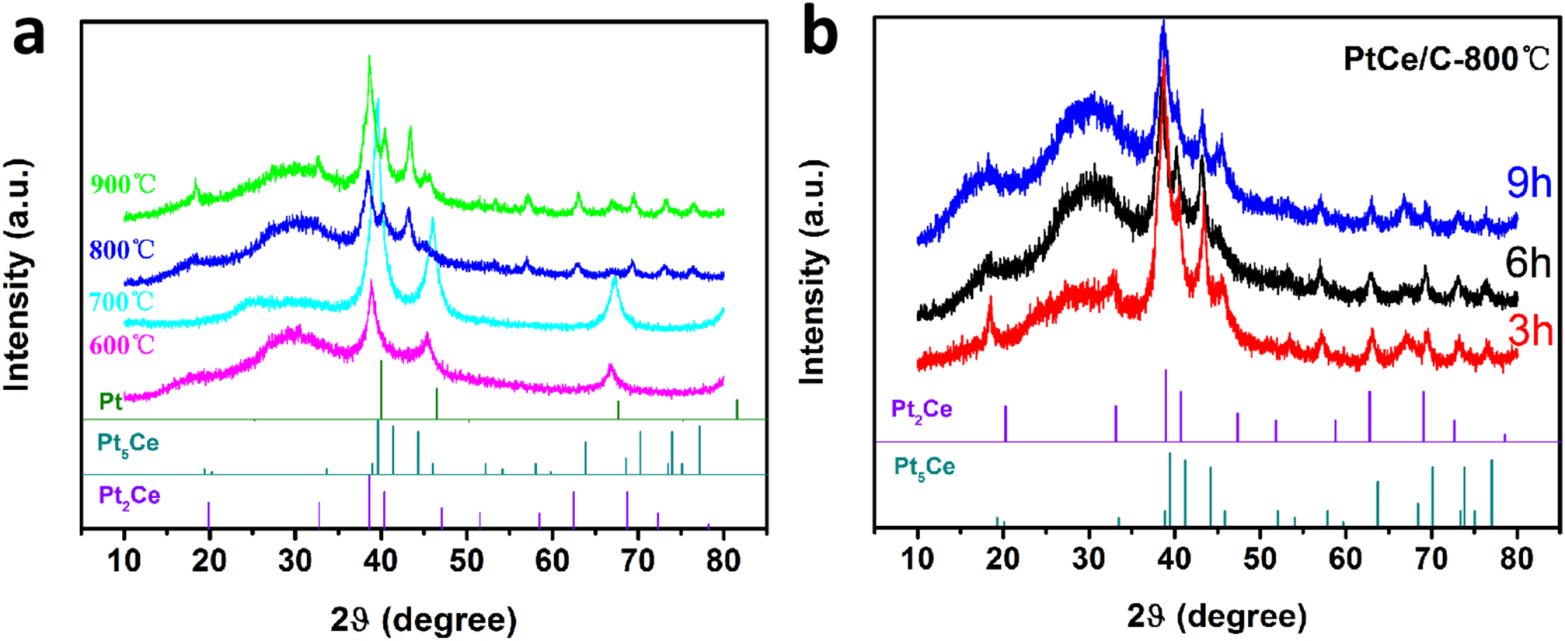
(**a**) XRD patterns of PtCe/C electrocatalysts under different temperatures and (**b**) XRD patterns of PtCe/C-800 ℃ sample with different annealing time.

XRD analysis technique was performed to verify the crystal phase of Pt-Ce nanoalloy. Figure 1a shows XRD pattern of the Pt-Ce nanoalloy under four different annealing temperatures. Three main peaks at 39.76°, 46.24° and 67.45° were indexed as (111), (200) and (220) crystal faces respectively, for crystalline face centered cubic (fcc) Pt (PDF 04-0802). The obvious shifts of Pt peaks are observed. It is most likely due to the CeCl_3_ was firstly reduced to Ce element and then Ce atoms diffused into the Pt crystal to expand the lattice of Pt. Additionally, for the two samples with the annealing temperatures of 800℃ and 900℃, there are many weaker peaks. After matching with the standard PDF files, we found that some of these peaks originate from Pt_2_Ce (PDF #17-0010), some from Pt_5_Ce (PDF #17-0071), but the rest peaks locate between the two standard card. Interestingly, the other pattern in Fig. 1b indicates the different annealing time hardly affected the Pt–Ce nanoalloy phase structure under annealing temperature 800 ℃. The average sizes of all samples were calculated by Scherrer’s equation. The results in Table 1 manifest the size of the Pt–Ce nanoalloy is significantly larger than that of Pt particles and the particle sizes increase with increasing annealing temperatures.

**Table 1:** Comparison of particle size of PtCe/C catalysts determined from Scherrer’s equation and statistic data through TEM, respectively.

Catalysts	FWHM	Cos(θ)	Particle size (nm)	TEM (nm)
PtCe/C-900 ℃	0.0033	0.9437	14.66	–
PtCe/C-800 ℃	0.0182	0.9412	10.55	10.21
PtCe/C-700 ℃	0.0174	0.9431	8.36	7.82
PtCe/C-600 ℃	0.0176	0.9408	7.46	6.48

**Figure 2:**
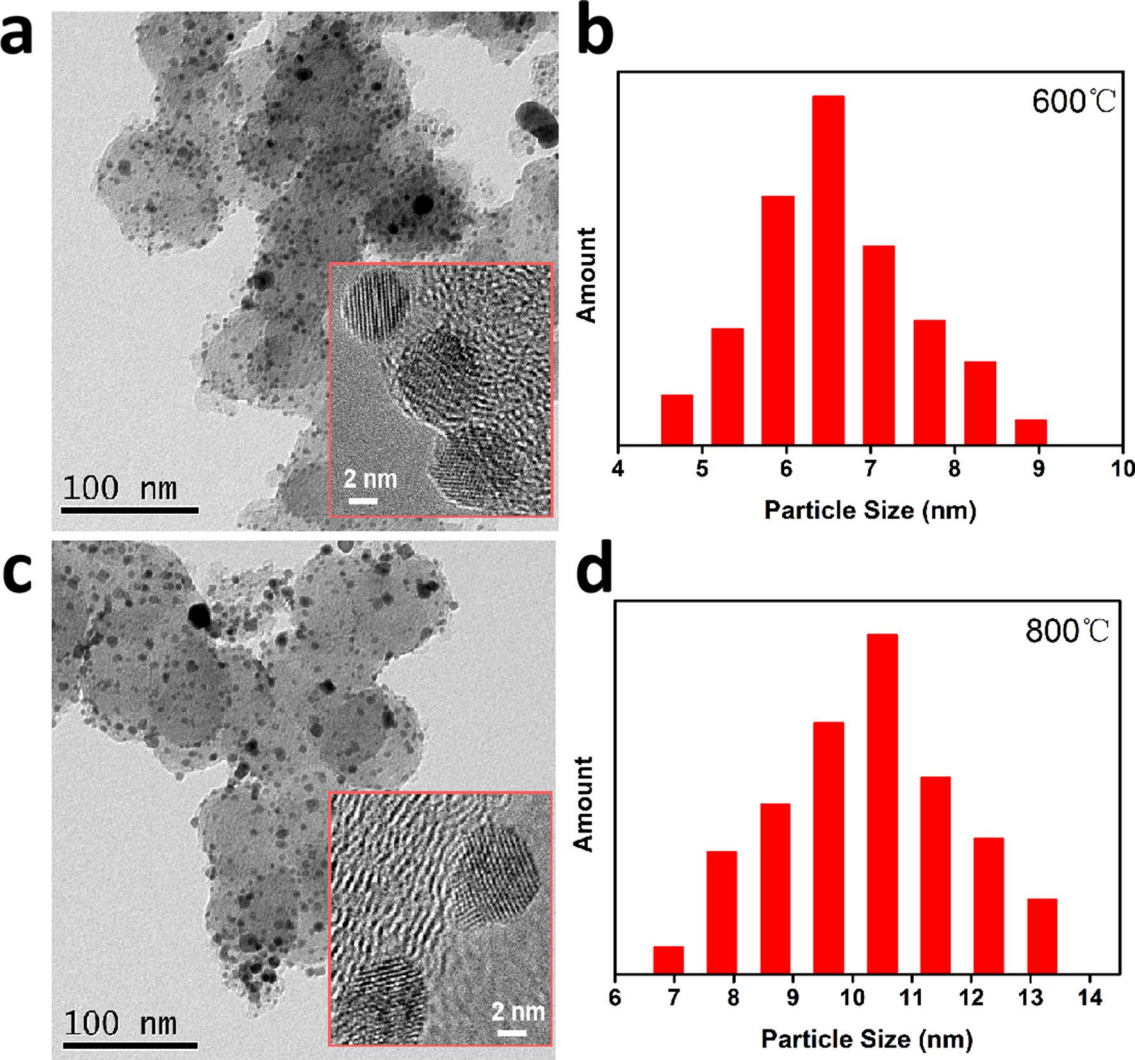
(**a**) TEM of PtCe/C-600 ℃ sample, the inset (red rectangular box) is the HRTEM and (**b**) Particle size distribution of PtCe/C-600 ℃ sample. (**c**) TEM of PtCe/C-800 ℃ sample, the inset (red rectangular box) is the HRTEM and (**d**) Particle size distribution of PtCe/C-800 ℃ sample.

TEM was used to analyze the particle size, the dispersion and the structure of Pt–Ce nanoalloy. Figure 2 shows TEM images and size distribution histograms of the Pt–Ce nanoalloy catalysts prepared under the annealing temperatures of 600 °C and 800 °C. It can be seen that the particle sizes of Pt–Ce alloy catalysts prepared at different reduction temperatures have wide distribution. Correspondingly, the size distribution histograms in Fig. 2b,d also clearly proved this. The size of Pt–Ce alloy catalysts which ranged from 4.5 to 9.5 nm with average size of 5.0 nm at annealing temperature 600 °C. Similarly, the size distribution changed from 6.5 nm to 13.5 nm with average size of 7.0 nm when the annealing temperature increased to 800 °C. In addition, the results have also been obtained for the samples which were annealed under 700 °C and 900 °C. The corresponding statistics of Pt–Ce alloy catalysts were listed in the Table 1. It obviously displays the particle sizes of all Pt–Ce alloy catalysts obtained from the TEM are agreed well with that from XRD.

**Figure 3:**
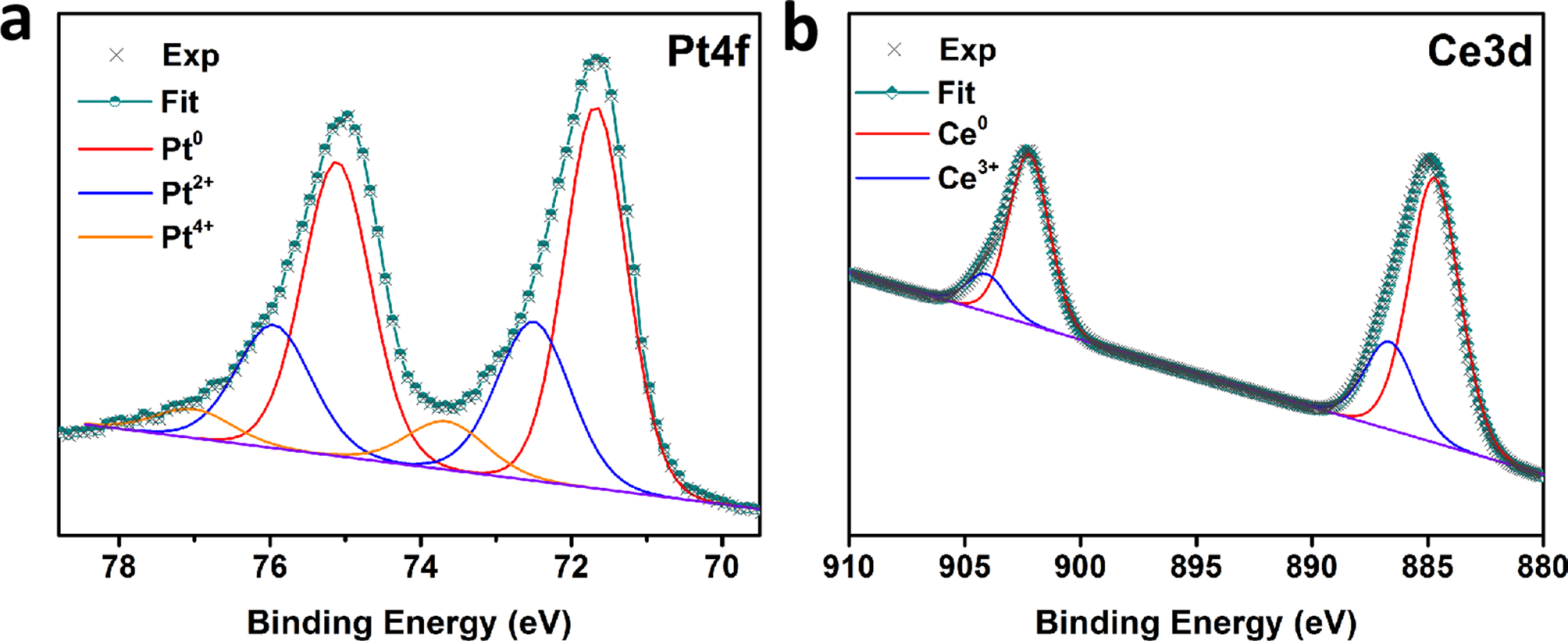
XPS spectra of (**a**) Pt 4f and (**b**) Ce 3d for Pt-Ce/C-800 ℃ alloy catalyst.

To further analyze the electronic structure and surface composition of Pt-Ce alloy catalyst, XPS spectrum of PtCe/C-800 ℃ sample was carried out. As shown in Fig. 3a, the Pt 4f XPS spectrum can be split into three pairs of peaks: the strongest couple emerged at 71.69 eV (Pt 4f_7/2_) and 75.21 eV (Pt 4f_5/2_) corresponds to Pt^0^ and the stronger pair presented at 72.52 eV (Pt 4f_7/2_) and 75.98 eV (Pt 4f_5/2_) is derived from Pt^2+^, while the weakest pair located at 73.68 eV (Pt 4f_7/2_) and 77.04 eV (Pt 4f_5/2_) is assigned to Pt^4+^ species^21^. Meanwhile, the Ce 3d XPS spectra in Fig. 3b is disintegrated into two couples of peaks: one pair located at 884.59 eV (Ce 3d_3/2_) and 902.33 eV (Ce 3d_5/2_) is attributed to metal Ce^22,23^, while the couples at 886.65 eV (Ce 3d_3/2_) and 904.17 eV (Ce 3d_5/2_) were stemmed from Ce^3+^. According to the data, the vast majority of metallic Ce^0^ was the dominant state of cerium for Pt–Ce alloy catalyst. A trace amount of cerium oxide remained, it is mainly because cerium, as one of the most active elements among the rare earth metals, is particularly easy to be oxidized to Ce^3+^ even under hypoxic atmosphere.

The elemental composition of Pt–Ce nanoalloy was detected by the energy dispersive X-ray spectroscopy (EDX) and inductively coupled plasma optical emission spectrometer (ICP-OES). As listed in Table 2, it reveals that the Pt contents are higher than 97% when the annealing temperature below 700 °C. It means that a small amount of Ce have been reduced and diffused into the Pt nanoparticles. The Ce contents increased to 13.7% and 14.5% when the reduction temperature increased to 900 °C and 800 °C, the ratio is higher than the Ce contents in Pt_5_Ce (12.5%) but lower than that in Pt_2_Ce (26%). It suggests that the alloy catalysts are mixed alloy phases of Pt_2_Ce and Pt_5_Ce. This is agree well with the results of XRD analysis.

**Table 2:** Comparison of average weight percentage of Ce metal obtained from EDX and ICP-OES metal analysis, respecrively.

Catalyst	Ce average wt (%)	
	EDX	ICP-OES
PtCe/C-900 ℃	13.75	12.83
PtCe/C-800 ℃	14.52	13.64
PtCe/C-700 ℃	3.13	2.48
PtCe/C-600 ℃	1.46	1.95

**Figure 4:**
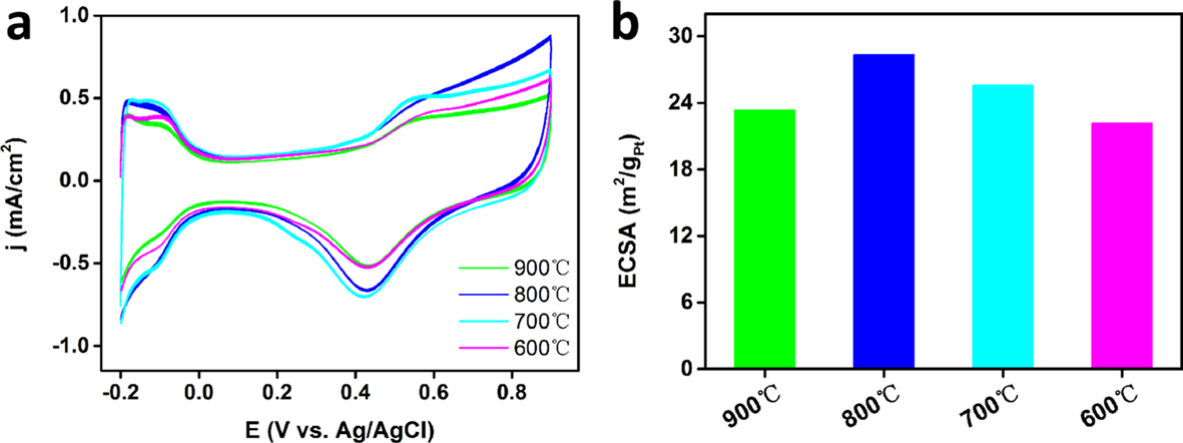
(**a**) Cyclic voltammetry curves and (**b**) ECSA bar graph of PtCe/C catalysts under different annealing temperatures.

CV tests were performed in an aqueous N_2_-saturated 0.1 M HClO_4_ solution with a scan rate of 100 mV s^−1^. As presents in Fig. 4a, a well-defined under potential deposition Hydrogen domain (Hupd) between − 0.20 and − 0.05 V was exhibited. With the annealing temperature increases, the current density increases non-linearly. Although the current density reaches the maximum when annealing temperature reaches 700 ℃ or 800 ℃, there are no apparent changes between the four samples. At the same time, Fig. 4b shows the electrochemical active surface area (ECSA) of the four Pt–Ce nanoalloy catalysts, which determined by measuring the charge collected in the hydrogen desorption region and assuming a value of 210 µC cm^−2^ for a monolayer hydrogen adsorption. Obviously, the ECSA of the four samples changed in the range of 24 m^2^ g_Pt_^−1^ to 30 m^2^ g_Pt_^−1^, which smaller than that of Com Pt/C (70.63 m^2^ g_Pt_^−1^) catalyst tested separately as a reference. And the variation trend of ECSA is agree well with the change trend of Ce concentration in the Pt–Ce nanoalloy catalysts. It further indicates the higher Ce concentration in Pt–Ce nanoalloy catalysts will enhance the ECSA of the nanoally.

**Figure 5:**
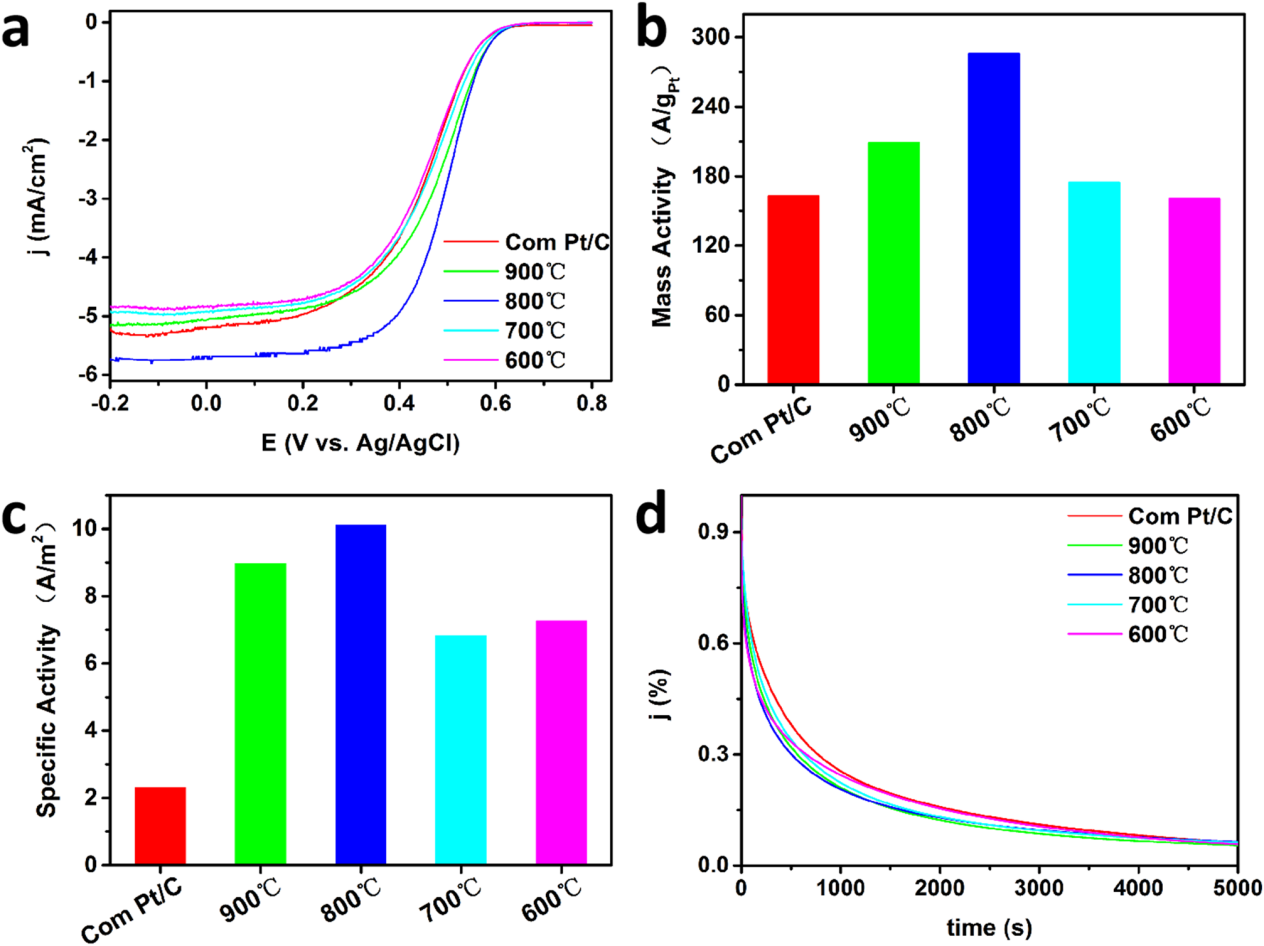
(**a**) Linear sweep voltammetry curves with a scan rate of 10 mV s^−1^, (**b**) mass activity, (**c**) specific activity, (**d**) chronoamperometry curves of Com Pt/C and synthesized PtCe/C catalysts under different annealing temperatures.

Linear sweep voltammetry (LSV) measurements were also collected to confirm the ORR activities of the Pt-Ce alloy catalysts and Com Pt/C. Figure 5a shows the LSV curves of the catalysts in solution of O_2_-saturated 0.1 M HClO_4_ at a scan rate of 10 mV s^−1^ at 1,600 rpm. The half-wave potential are 0.45, 0.45, 0.45, 0.50 and 0.48 V (vs. Ag/AgCl) for Com Pt/C, PtCe-600 ℃, PtCe-700 ℃, PtCe-800 ℃ and PtCe-900 ℃ respectively. The mass activity (MA) in Fig. 5b of Com Pt/C, PtCe-600 °C and PtCe-700 ℃ are about 160 A g_Pt_^−1^ (at 0.5 V vs. Ag/AgCl), while that of PtC-800 ℃ and PtCe-900 ℃ increase to 286 and 209 A g_Pt_^−1^ respectively. It indicates that Pt–Ce alloy-structure catalysts can effectively improve the ORR activity, and the catalytic activity increase with the increasing of the Ce content. Correspondingly, Fig. 5c illustrates that all Pt–Ce catalysts have higher specific activity (SA) than Com Pt/C, and the SA also increases with increasing Ce content. In addition, the electrochemical stability of all samples and Com Pt/C was studied by Chronoamperometry (CA) technique in 0.1 M HClO_4_ at 0.5 V (vs. Ag/AgCl). As shown in Fig. 5d, all the curves show a sharp initial current drop in the first 300 s and then decay very slowly until 5000 s. In contrast to the previous reports on Pt_5_M polycrystalline^24,25^ (M = La, Ce or Gd etc.), the Ce doping did not significantly improve the stability of the Pt–Ce alloy catalyst in this work. This is mostly because the reduced Ce atoms will diffuse from the edge of the Pt particles into the nucleus. With the diffusion increases, the concentration of Ce atoms at the edge of the Pt particle gradually decrease, eventually a Ce skin is formed.

## Conclusions

In this work, We have synthesized Pt-Ce nanoalloy by hydrogen reduction method at various reduction temperatures. By performing XRD, TEM, XPS and ICP-OES analysis, we studied in detail the composition and structure of the synthesized Pt–Ce nanoalloy. The results are consistent with the occurrence of CeCl_3_ reduction and Ce atoms diffusion with the annealing temperature reaches 800 °C, which results in a mixed alloy phases of Pt_2_Ce and Pt_5_Ce. The electrochemical results demonstrate that the Pt–Ce alloy catalysts can effectively improve the ORR activity and the catalytic activity increases with increasing Ce concentration.

## Methods

### Material preparation

Firstly, the heat-treated Pt/C nanoparticles mixed with CeCl_3_ which was dissolved in anhydrous acetonitrile (99.8%, Sigma-Aldrich, < 10 ppm of H_2_O). The mixture was stirred overnight and then dried at 70 °C. After that, a dry powder was obtained with a ratio of Pt:Ce of 1.89:1. The dry powder was moved into the tube furnace. The reactor was filled with nitrogen at 100 SCCM for 30 min. then with hydrogen for 60 min at 100 SCCM. After that, the temperature was increased to set point (600, 700, 800 and 900 °C) with 5 °C/min and kept 3 or 6 h then cooled down to room temperature under 100 SCCM hydrogen flow. Prior to any characterization, the as-synthesized Pt-Ce nanoalloies were acid-washed with 0.1 M HClO_4_ and water-washed three times to remove any unreacted traces of precursor and Ce_2_O_3_ layer on the surface of the samples. Commercial Pt/C (20 wt%, Sigma-Aldrich) was used for comparison of the activity.

### Material characterizations

The crystalline structure of the PtCe/C catalysts was verified by X-ray diffraction (Bruker D2 PHASER diffraction) with Cu Kα radiation (λ = 1.5406 Å). The morphological, size, distribution and composition of Pt-Ce/C alloy catalysts was investigated by transmission electron microscopy (TEM, FEI Tecnai G2 F20) with an acceleration voltage of 200 kV attached to energy-dispersive X-ray spectroscopy analyzer for elemental analysis. The electronic structure and surface composition of the synthesized Pt–Ce/C alloy catalysts was performed by X-ray photoelectron spectroscopy (XPS, Termo Fisher Kα, USA). The concentration of elements Pt and Ce in Pt–Ce/C catalysts was also detected by Inductively Coupled Plasma Optical Emission Spectrometer (ICP-OES, Leeman Labs Prodigy 7 instrument).

### Preparation of electrode

The catalyst ink was prepared by dispersing a certain amount of catalyst power into a mixed solution of ethanol (79.98 v/v%), deionized water (20 v/v %) and 5 wt% of Nafion solution (0.02v/v%, DuPont, US), followed with sonication for 30 min. The prepared catalyst slurries were placed on a surface of a polished glassy carbon disk electrode (0.196 cm^2^) and then dried by air flow at room temperature. The catalyst loadings were equal to 0.102 mg cm^−2^.

### Electrochemical measurements

Electrochemical activity tests were carried out by using a CHI 760E electrochemical workstation (CHI Instruments Co.,Shanghai, China) with typical three-electrode system. The three-electrode system including glassy carbon electrode as a working electrode, platinum sheet as a counter electrode and Ag/AgCl as the reference electrode. Rotating disk electrode (RDE) voltammetry was conducted on a Pine Research Instrument (AFMSRCE, USA) regulated rotation speed. Cyclic voltammetry (CV) tests were measured in 0.1 M HClO_4_ aqueous solution with a scan rate of 100 mV·s^−1^. Linear sweep voltammetry (LSV) curves were obtained using a rotation disk electrode at 1,600 rpm with a scan rate of 10 mV·s^−1^ in 0.1 M HClO_4_. Chronoamperometry (CA) measurements were performed in 0.1 M HClO_4_ electrolyte solution for 5,000 s. Before each test procedure, the electrolyte was bubbled by N_2_ (or O_2_) gas flow for 30 min. All experiments were carried out in a sealed condition.
